# Psychological states mediate the relationship between sleep quality and frailty among older adults

**DOI:** 10.3389/fpsyg.2025.1691997

**Published:** 2025-11-12

**Authors:** Huabin Su, Liuxiang Wei, Yanyan Lin, Caiyou Hu, Yuan Lv, Hua Li, Xianghua He, Di Zhang, Xiaolin Ni

**Affiliations:** 1 Jiangbin Hospital of Guangxi Zhuang Autonomous Region, Nanning, China; 2 Dongge Community Health Service Center, Nanning, China; 3 State Key Laboratory of Respiratory Health and Multimorbidity, Institute of Basic Medical Sciences, Chinese Academy of Medical Sciences and Peking Union Medical College, Beijing, China

**Keywords:** older adults, frailty, sleep quality, psychological state, moderated mediation

## Abstract

**Objective:**

Frailty prevalence is high among older adults. While the association between poor sleep quality and frailty is established, the mediating role of psychological states (anxiety/depression) and the moderating influence of multidimensional factors (physiological conditions, dietary habits, and lifestyle factors) on this pathway remain unclear. This study aimed to examine the mediating effects of anxiety and depression on the sleep quality-frailty link and identify moderators of the sleep quality-psychological state relationship among community-dwelling older adults.

**Methods:**

A cross-sectional survey was conducted among 900 adults aged ≥60 years in Dongge Community, Nanning, Guangxi. Sleep quality, anxiety, depression, and frailty were assessed using the Pittsburgh sleep quality index (PSQI), hospital anxiety and depression scale (HADS-A/HADS-D), and FRAIL scale, respectively. Data on physiological conditions, dietary habits, and lifestyle factors were collected via questionnaire. Parallel mediation and moderated mediation analyses were performed.

**Results:**

Mean scores were: HADS-A = 1.00 ± 1.76, HADS-D = 1.32 ± 2.11, PSQI = 6.41 ± 3.22, and FRAIL = 0.96 ± 1.05. Parallel mediation analyses confirmed that both anxiety (*B* = 0.029, 95%CI [0.004, 0.060]) and depression (*B* = 0.018, 95%CI [0.001, 0.042]) partially mediated the association between poor sleep quality and frailty, collectively accounting for 23.50% of the total effect. Moderated mediation analyses revealed that bodily pain, specific dietary habits (intake frequency ≥5 times/week of milk, soybeans, and fish/meat/eggs), and daily outdoor exercise duration (≥30 min) significantly moderated the strength of the mediating pathway through anxiety (i.e., the “sleep quality → anxiety” link).

**Conclusion:**

Anxiety and depression significantly mediate the relationship between poor sleep quality and frailty in older adults. Crucially, this mediating pathway via anxiety is modifiable, being attenuated by the absence of bodily pain, frequent consumption of key protein-rich foods, and regular outdoor exercise. These findings highlight potential targets for multi-faceted interventions aimed at mitigating frailty risk by improving sleep and psychological well-being in aging populations.

## 1 Introduction

Frailty is a progressive, age-related decline in the functioning of multiple physiological systems. This decline reduces intrinsic capacity and increases vulnerability, which in turn raises the risk of adverse health outcomes, such as fractures, hospitalizations, co-morbidities, and mortality (Cesari et al., 2017; Dent et al., 2019; Vermeiren et al., 2016; Hoogendijk et al., 2019). As an emerging global health burden, frailty demonstrates high prevalence among older populations. The pooled global prevalence of frailty is 43.4 cases per 1,000 person-years, while the overall prevalence of frailty and pre-frailty in Chinese community-dwelling older adults is 10.1 and 43.9%, respectively, which is significantly higher than the global average (Ofori-Asenso et al., 2019; Zhou et al., 2023). Consequently, it is important to explore the interventional risk factors for frailty in Chinese older adults to reduce the individual and public health burden.

Previous studies have suggested that sleep quality is significantly associated with a debilitating state in old age (Balomenos et al., 2021; Alqahtani, 2021). Notably, sleep quality problems are prevalent in the elderly population and partly stem from difficult-to-intervene factors such as physiologic aging (Pappas and Miner, 2022). Sleep quality problems and psychological states (anxiety and depression) often coexist (Chellappa and Aeschbach, 2022; Baglioni et al., 2011), and psychological states themselves are not only important risk factors for frailty, but may also reduce the likelihood of recovery in frail older adults (Mutz et al., 2022). Previous studies have demonstrated associations among sleep quality, psychological states, and frailty. However, a critical gap remains in understanding whether psychological states, specifically anxiety and depression, serve as mediators in the relationship between sleep quality and frailty. Therefore, we propose the first hypothesis: anxiety and depression mediate the relationship between sleep quality and frailty.

Aging is a natural stage in the life course, and the frailty state accompanying it is often manifested as multidimensional decline in physiological, psychological and social functions. In the process of aging, adopting effective strategies to maintain or improve the quality of life is an important goal of achieving healthy aging. Existing studies have pointed out that specific dietary patterns (such as the Mediterranean diet) and healthy lifestyles (such as regular physical exercise) may help alleviate the adverse health effects of frailty (Ni Lochlainn et al., 2021; Sanchis-Soler et al., 2025). Additionally, frailty is often accompanied by a series of clinically relevant risks, such as chronic pain, falls, and malnutrition (Wang et al., 2022). However, the extent to which multidimensional physiological indicators, dietary habits, and lifestyle factors-considered as intervening variables-moderate the association between sleep quality and psychological states remains unclear. Clarifying this mechanism is of great significance for formulating precise intervention strategies. Accordingly, we propose the second hypothesis: the association between sleep quality and psychological state are moderated by specific physiological conditions, dietary habits, and lifestyle factors.

**Figure 1:**
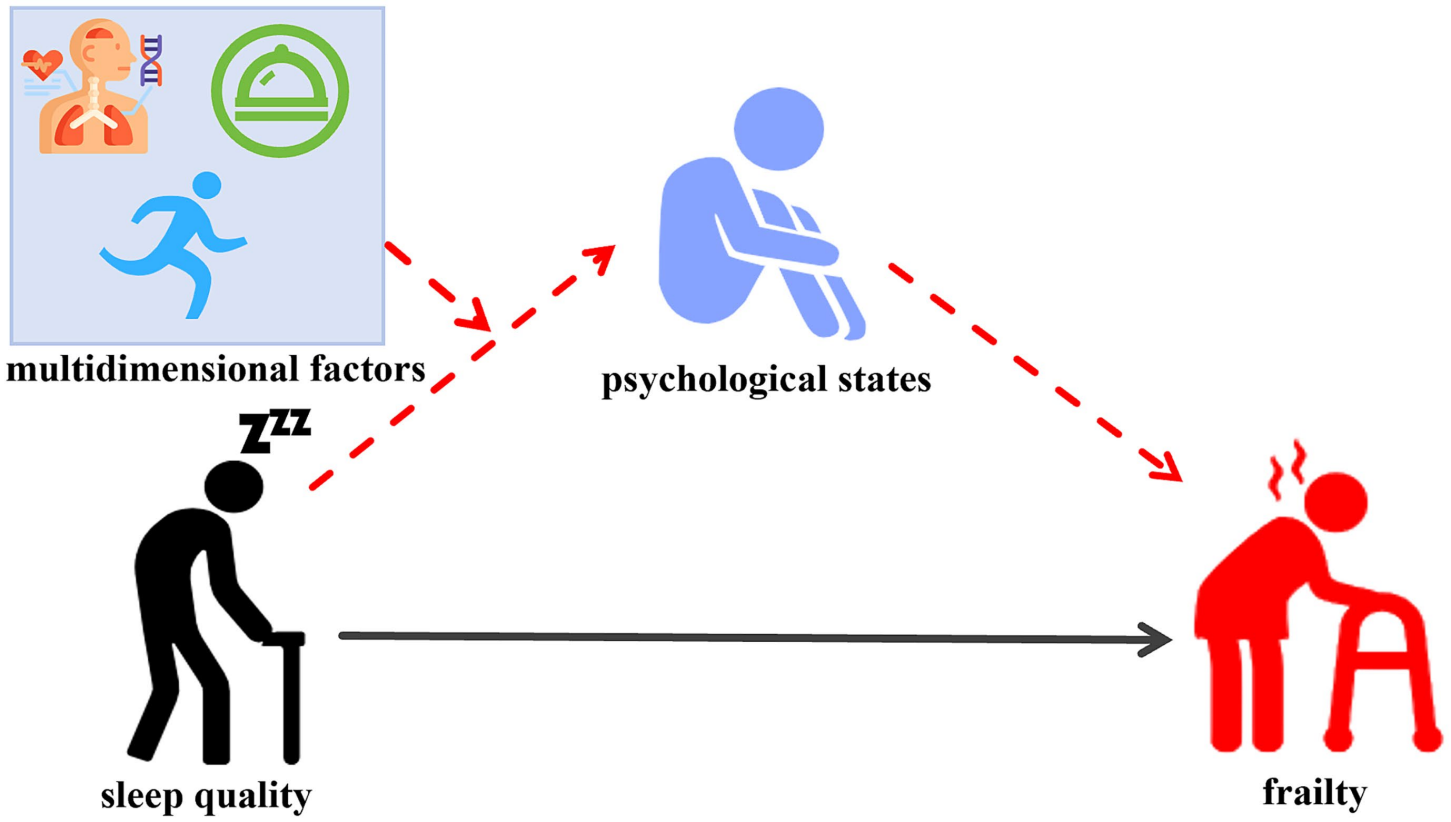
Illustration of the proposed moderated-mediation conceptual model. This study aimed to examine whether psychological states (anxiety and depression) mediate the relationship between sleep quality and frailty in older adults, along with examining how multidimensional factors, including physiological conditions, dietary habits, and lifestyle factors, moderate the association between sleep quality and the psychological states. Solid lines represent established findings from the literature, whereas dashed lines indicate the novel hypotheses tested in this study. Psychological states: anxiety and depression. Multidimensional factors: physiological conditions, dietary habits, and lifestyle factors. The red dotted line indicates the hypothesis to be proven.

The correlation hypothesis model is illustrated in the conceptual framework (Figure 1). This model integrates established findings from the literature (solid lines) with the novel hypotheses tested in this study (dashed lines). It illustrating the hypothesized relationships between sleep quality, psychological states, multidimensional factors, and frailty.

This study aimed to examine whether psychological states (anxiety and depression) mediate the relationship between sleep quality and frailty in older adults, along with examining how multidimensional factors, including physiological conditions, dietary habits, and lifestyle factors, moderate the association between sleep quality and the psychological states.

## 2 Methods

### 2.1 Study design and participants

The cross-sectional study was conducted from July to December 2023 in Dongge Community, Nanning City, Guangxi Zhuang Autonomous Region. Inclusion criteria: (1) age ≥60 years; (2) completion of the corresponding scale assessment; (3) complete assessment data available; (4) agreement to participate in this study and completion of follow-up. Medical exclusion criteria: (1) patients with chronic diseases such as hypertension, diabetes mellitus, chronic gastritis, stroke, dementia, epilepsy, cancer; (2) Medications use for over 12 months; (3) communication disorders. Methodological exclusion criteria: (1) incomplete questionnaire data were considered missing date. A list wise deletion approach was therefore applied to handle these cases; (2) univariate outliers were identified and excluded from the analysis to ensure data integrity. Specifically, values for height and weight that exceeded ±3 standard deviations from the mean were considered outliers and removed. This study adhered to the principles of the Declaration of Helsinki. The Ethics Committee of the Jiangbin Hospital of Guangxi Zhuang Autonomous Region approved the study protocol (KY-GZR-2022-03). All study participants provided written informed consent. All data were anonymized upon collection, and were accessible only to the research team for analysis purposes.

### 2.2 Measures

#### 2.2.1 Sociodemographic and clinical variables

Sociodemographic and clinical data were collected using a comprehensive paper-based questionnaire that included the following domains: demographic characteristics (gender, age, marital status), physiological conditions (height, weight, bodily pains, falls history, and recent weight change), dietary habits [daily meal frequency, frequency of milk consumption, soybean, and fish/meat/eggs (≥5 times per week)], and whether drinking water reaches 1,200 mL daily (self-report diary), and lifestyle factors (whether doing outdoor exercise for at least 30 min daily, smoking history and drinking history). Body mass index (BMI) was calculated as weight in kilograms divided by height in meters squared (kg/m^2^).

#### 2.2.2 Clinical variables

We separately measured participants’ sleep quality, anxiety, depression, and frailty via the Pittsburgh sleep quality index (PSQI) (Buysse et al., 1989), hospital anxiety and depression scale (HADS) (Zigmond and Snaith, 1983), and fatigue, resistance, ambulation, illness, and loss of weight (FRAIL) scale (Abellan van Kan et al., 2008).

### 2.3 Questionnaires

#### 2.3.1 The Pittsburgh sleep quality index (PSQI)

This study used the Pittsburgh sleep quality index (PSQI) to assess the sleep quality of older adults. Developed by Buysse et al. (1989), the PSQI is a questionnaire consisting of 19 self-rated items and 5 partner-rated items. Only the 19 self-rated items are scored. These 19 items form seven components, including subjective sleep quality, sleep latency, sleep duration, habitual sleep efficiency, sleep disturbances, use of sleeping medication, and daytime dysfunction. The total score of PSQI is the sum of scores from the seven components, which ranges from 0 to 21. A score of more than five indicates poor sleep quality, with higher scores reflecting worse sleep quality. The PSQI has good applicability among Chinese elderly community residents, and the Cronbach’s alpha coefficient was 0.680 (Zhang et al., 2020).

#### 2.3.2 Hospital anxiety and depression scale (HADS)

The hospital anxiety and depression scale (HADS) was developed by Zigmond and Snaith (1983) as a screening tool to identify possible and probable cases of anxiety and depression among patients in non-psychiatric hospital clinics (Zigmond and Snaith, 1983). HADS consists of two subscales. The symptoms of anxiety and depression were separately screened by the anxiety subscale (HADS-A) and the depression subscale (HADS-D). The total score of HADS-A and HADS-D was 21 points, with a score of ≥8 indicating the presence of anxiety and depression symptoms. Meanwhile, the higher scores of HADS-A and HADS-D represent the greater symptom levels of anxiety and depression. The Cronbach’s alpha coefficient for HADS ranges from 0.820 to 0.900 (Smarr and Keefer, 2011).

#### 2.3.3 Fatigue, resistance, ambulation, illness, and loss of weight (FRAIL)

Frailty was assessed via the fatigue, resistance, ambulation, illness, and loss of weight (FRAIL) scale. Proposed by experts from the International Association of Nutrition and Aging in 2008, the FRAIL scale comprises five items: fatigue, resistance, ambulation, illnesses, and weight loss (Abellan van Kan et al., 2008). The total score ranges from 0 to 5 with higher scores indicating a greater likelihood of frailty. The FRAIL scale was simple to operate and has been demonstrated to be a reliable and valid tool for screening frailty among the elderly individuals in China (Dong et al., 2018; Dent et al., 2017).

### 2.4 Statistical analysis

Data analysis was carried out using SPSS version 27.0 and PROCESS version 4.0. All statistical graphs were generated using GraphPad Prism (version 10.1.2) and Adobe Illustrator (version 2024). The final figure layout and formatting were completed using Adobe Illustrator (version 2024). Descriptive statistics and Spearman correlation analysis were calculated through SPSS 27.0. Continuous variables are presented as mean ± standard deviation; categorical variables are presented as frequency (percentage). Multicollinearity among variables was assessed using variance inflation factors (VIF exceeding 5 to 10 indicates significant multicollinearity) (Kim, 2019). Normality was examined using the Shapiro–Wilk test and visual inspection of Q-Q plots. Homoscedasticity was evaluated using the Breusch–Pagan test. As some variables deviated from normality, spearman’s rank correlation analysis was employed for correlation analyses. Prior to conducting the parallel mediation effects and moderated mediation analysis, continuous predictor variables were standardized into *z*-scores by subtracting the sample mean and dividing by the sample standard deviation for each variable. Categorical variables remained unstandardized. Model 4 and Model 7 of PROCESS version 4.0 were used to examine parallel mediation effects and moderated mediation effects, respectively (Igartua and Hayes, 2021). For all mediation analyses and moderated mediation analyses, the bias-corrected 95% confidence interval (CI) was calculated with a bootstrapping approach (with 5,000 bootstrap samples). If the 95%CI of the indirect effects excludes zero, it indicates the mediating effect was significant. Likewise, if the 95%CI of the interaction and moderated mediation index excludes zero, the moderated mediation effect proves to be statistically significant. All models were controlled for covariates (age, gender, marital status). The statistical significance was set at *p* < 0.05 (two-tailed).

## 3 Results

### 3.1 Characteristics of the participants

**Figure 2:**
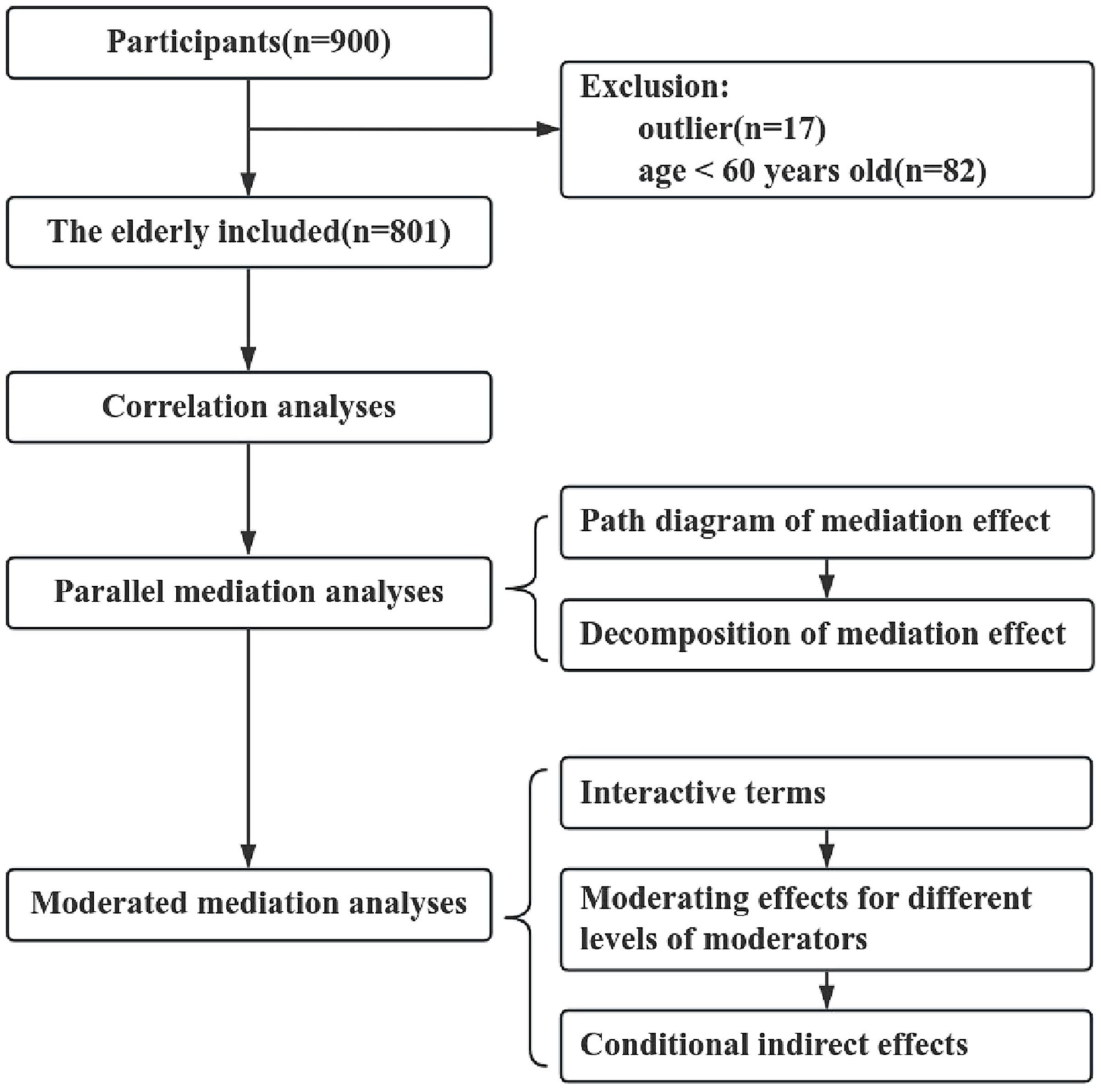
Flow diagram of the selection of participants and study design.

Self-report questionnaires were administered to 900 participants recruited via random sampling. Finally, a sample of 801 participants was included in this study. Exclusions were due to univariate outliers (*n* = 17) and age under 60 years (*n* = 82) (Figure 2). The mean age of the 801 participants was 72.1 ± 5.4 years. Among these participants, 376 (46.9%) were male and 677 (84.5%) were married. The majority of participants reported no bodily pain (52.7%), no fall history (86.4%), loss of weight (51.9%), and eating three times a day (64.9%). Most of the participants were able to intake milk (65.3%), soybean (71.8%), and fish/meat/eggs (87.1%) at least five times per week. 606 (75.7%) participants did outdoor exercise for at least 30 min daily, and 286 (35.7%) participants drank water ≥1,200 mL daily. Most participants had no history of smoking (88.1%) and drinking (87.3%). The average scores for HADS-A, HADS-D, PSQI, and FRAIL were 1.00 + 1.76, 1.32 ± 2.11, 6.41 ± 3.22, and 0.96 ± 1.05, respectively. The other relevant information is shown in Table 1.

**Table 1:** Demographic, physical condition, dietary habits, lifestyle information, and questionnaires characteristics of participants.

Characteristics	Mean ± SD or *n* (%)
Age	72.1 ± 5.4
Gender	
Male	376 (46.9)
Female	425 (53.1)
Marital status	
Single	124 (15.5)
Married	677 (84.5)
BMI	24.0 ± 3.3
Pain	
No	422 (52.7)
Yes	379 (47.3)
Fall	
No	692 (86.4)
Yes	109 (13.6)
Weight	
Unchange	385 (48.1)
Loss	416 (51.9)
Meal frequency	
Others	281 (35.1)
Three	520 (64.9)
Milk	
Sometimes or not	278 (34.7)
Always	523 (65.3)
Soybean	
Sometimes or not	226 (28.2)
Always	575 (71.8)
Fish/meat/eggs	
Sometimes or not	103 (12.9)
Always	698 (87.1)
Water	
<1,200 mL	515 (64.3)
≥1,200 mL	286 (35.7)
Exercise	
No	195 (24.3)
Yes	606 (75.7)
Smoking	
No	706 (88.1)
Yes	95 (11.9)
Drinking	
No	699 (87.3)
Yes	102 (12.7)
HADS-A	1.0 ± 1.76
HADS-D	1.32 ± 2.11
PSQI	6.41 ± 3.22
FRAIL	0.96 ± 1.05

Pain: bodily pains; fall: falls history; weight: recent weight change; meal frequency: daily meal frequency; milk, soybean, and fish/meat/eggs: intake of milk, soybean, and fish/meat/eggs five times per week; water: whether drinking water reaching 1,200 mL daily; exercise: whether doing outdoor exercise for at least 30 min daily; smoking: smoking history; drinking: drinking history; PSQI: sleep quality; HADS-A: anxiety; HADS-D: depression; FRAIL: frailty.

### 3.2 Correlation analysis

To mitigate multicollinearity concerns, variance inflation factor (VIF) diagnostics were conducted on key variables (Kim, 2019). As summarized in Supplementary Table S1, all VIF values remained below the threshold of 5, confirming absence of significant multicollinearity among independent variables.

**Figure 3:**
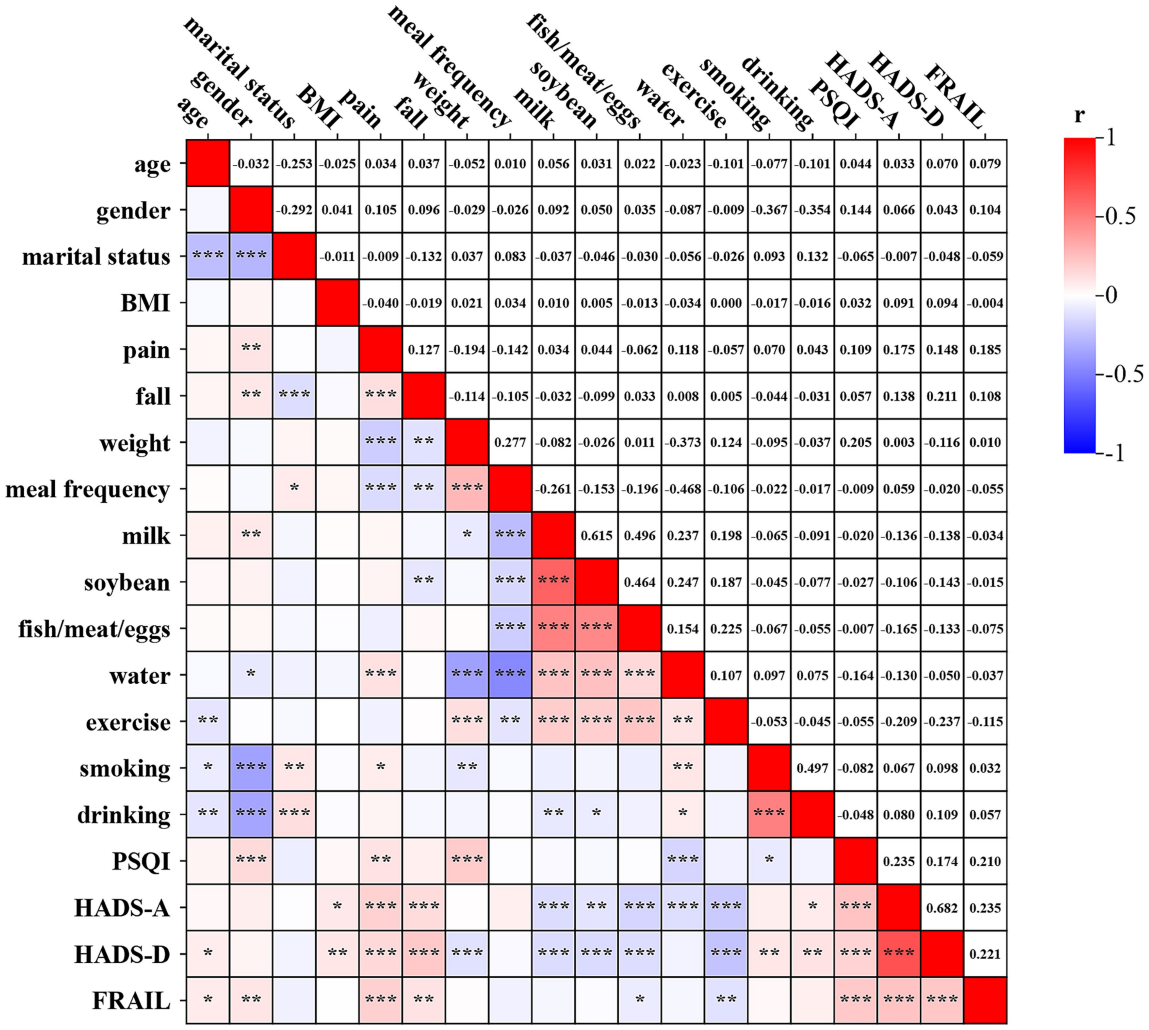
Spearman correlations among variables (*n* = 801). Pain: bodily pains; fall: falls history; weight: recent weight change; meal frequency: daily meal frequency; milk, soybean, and fish/meat/eggs: intake of milk, soybean, and fish/meat/eggs five times per week; water: whether drinking water reaching 1,200 mL daily; exercise: whether doing outdoor exercise for at least 30 min daily; smoking: smoking history; drinking: drinking history; PSQI: sleep quality; HADS-A: anxiety; HADS-D: depression; FRAIL: frailty. *p < 0.05; **p < 0.01; ***p < 0.001.

Bivariate analyses of 801 older adults revealed significant correlations (all *p* < 0.05): sleep quality exhibited positive associations with female sex, bodily pain, and recent weight changes, but inverse relationships with daily water intake and smoking history. Anxiety levels demonstrated positive correlations with bodily pain, fall history, recent weight change, and sleep quality, whereas inverse associations emerged with BMI, intake frequency for milk, soybean, and fish/meat/eggs, and daily outdoor exercise duration. Depression showed positive relationships with bodily pain, fall history, smoking history, drinking history, sleep quality, and anxiety, while correlating inversely with recent weight changes, daily meal frequency, intake frequency for milk, soybean, and fish/meat/eggs, and daily outdoor exercise duration. Frailty displayed positive correlations with age, BMI, bodily pain, fall history, sleep quality, anxiety, and depression, with daily outdoor exercise duration constituting its sole inverse correlate. The rest of the relevant information is shown in Figure 3.

### 3.3 Parallel mediation analyses

**Figure 4:**
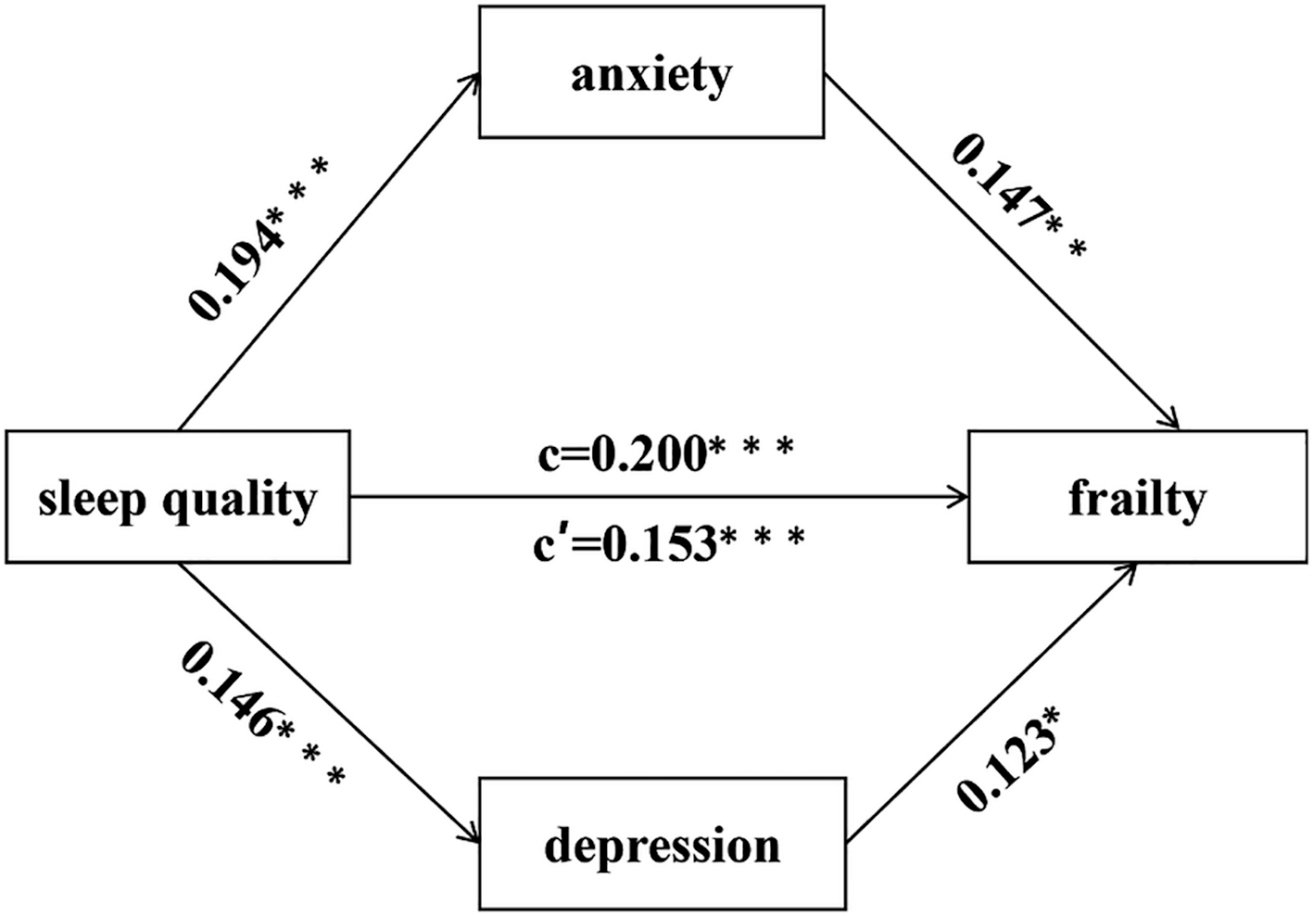
The parallel mediation effects of anxiety and depression. c = total effects; c’ = direct effects. *p < 0.05; **p < 0.01; ***p < 0.001. Adjusted based on gender, age, and marital status.

After adjusting for age, gender, and marital status, mediation effects were tested using Model 4 with sleep quality as the independent variable, frailty as the dependent variable, and anxiety and depression as the mediating variables. The results showed that sleep quality was a significant positive predictor of frailty (*B* = 0.200, *p* < 0.001) (Figure 4). The direct effect of sleep quality on frailty remained significant after adding the mediating variables (*B* = 0.153, *p* < 0.001) (Figure 4). Mediation analyses showed significant indirect effects for both anxiety (*B* = 0.029, 95%CI [0.004, 0.060]) and depression (*B* = 0.018, 95%CI [0.001, 0.042]) (Table 2). The total effect could be decomposed into a direct effect (*B* = 0.153, 95%CI [0.083, 0.224]) and a total indirect effect (*B* = 0.047, 95%CI [0.022, 0.077]), accounting for 76.50 and 23.50% of the total effect, respectively. The mediating effect of anxiety accounted for 14.50% of the total effect and the mediating effect of depression accounted for 9.00% of the total effect. However, the difference in the amount of indirect effects for anxiety and depression was not statistically significant (*B* = 0.011, 95%CI [− 0.029, 0.053]) (Table 2).

**Table 2:** The mediating effect decomposition table.

Effect	B	Boot SE	Boot 95%CI	Effect ratio
Total effect	0.200	0.036	0.130, 0.270	
Direct effect	0.153	0.036	0.083, 0.224	76.50%
Mediating effect	0.047	0.014	0.022, 0.077	23.50%
Anxiety	0.029	0.014	0.004, 0.060	14.50%
Depression	0.018	0.011	0.001, 0.042	9.00%
(C1)	0.011	0.021	−0.029, 0.052	/

(C1): Significant difference in the mediating effect. Effect ratio is defined as the direct effects or mediation effect divided by the total effect.

### 3.4 Moderated mediation analyses

**Figure 5:**
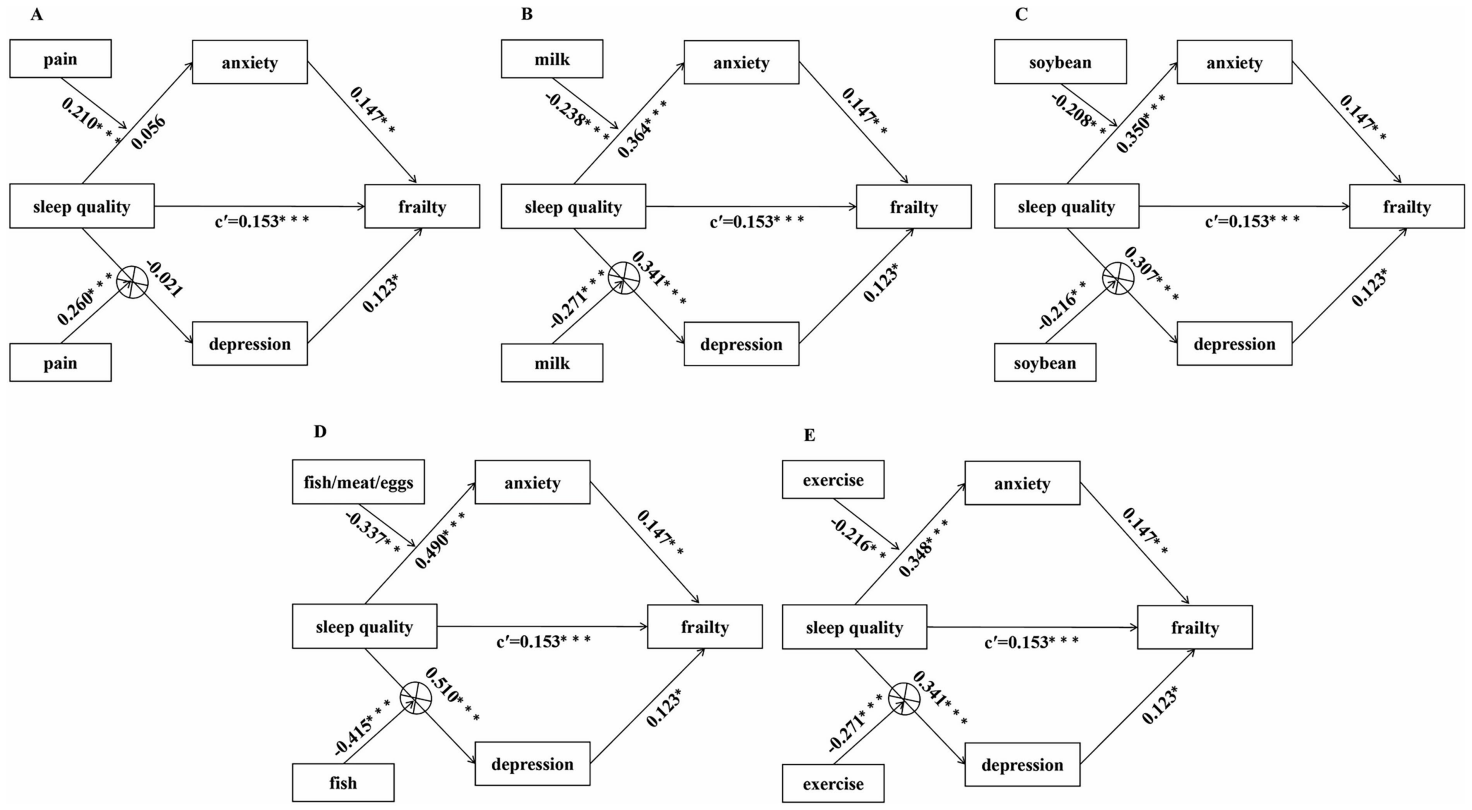
The moderated mediation path diagram. **(A)** The moderating effect of bodily pains on the mediation model; **(B)** the moderating effect of milk: intake of milk five times per week on the mediation model; **(C)** the moderating effect of soybean: intake of soybean five times per week on the mediation model; **(D)** the moderating effect of fish/meat/eggs: intake of fish/meat/eggs five times per week on the mediation model; **(E)** the moderating effect of exercise: whether doing outdoor exercise for at least 30 min daily on the mediation model. c’ = direct effects. *p < 0.05; **p < 0.01; ***p < 0.001. ⊗: The conditional indirect effects are not valid.

To further explore whether the mediating effects of anxiety and depression were moderated by specific physiological conditions, dietary habits, or lifestyle factors, the moderated mediating effects were tested using Model 7. After adjusting for gender, age, and marital status, the results (Figure 5) showed that bodily pains over the past years positively moderated the relationship between sleep quality and psychological states(anxiety: *B* = 0.210, *p* < 0.001; depression: *B* = 0.260, *p* < 0.001) (Figure 5A), while intake of milk (anxiety: *B* = −0.238, *p* < 0.001, depression: *B* = −0.271, *p* < 0.001) (Figure 5B), soybean(anxiety: *B* = −0.208, *p* < 0.01; depression: *B* = −0.216, *p* < 0.01) (Figure 5C), and fish/meat/eggs (anxiety: *B* = −0.337, *p* < 0.01; depression: *B* = −0.415, *p* < 0.001) (Figure 5D) five times per week and whether doing outdoor exercise for at least 30 min daily (anxiety: *B* = −0.216, *p* < 0.01; depression: *B* = −0.271, *p* < 0.001) (Figure 5E) negatively moderated the relationship between sleep quality and psychological states. In addition, changes in weight over the past 3 months positively moderated only the effect of sleep quality on depression. The remaining factors have no moderating effects (Supplementary Figure S1).

**Figure 6:**
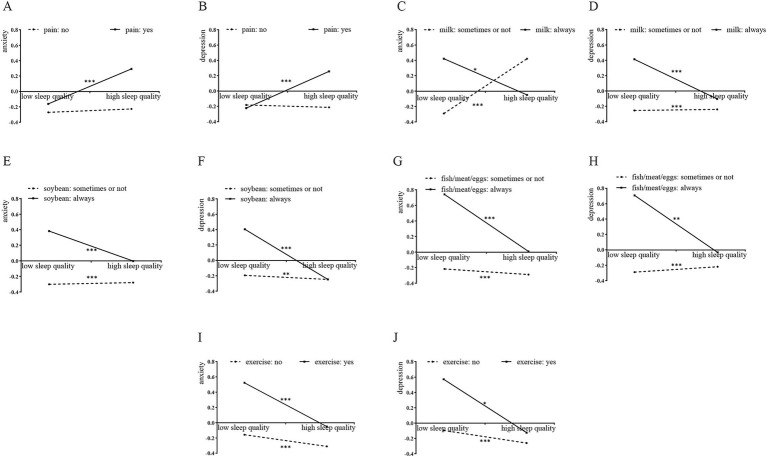
The simple slope plots with the multidimensional factors as a moderator. **(A)** Whether bodily pains act as a moderator of the connection between sleep quality and anxiety; **(B)** whether bodily pains acts as a moderator of the connection between sleep quality and depression; **(C)** intake of milk five times per week acts as a moderator of the connection between sleep quality and anxiety; **(D)** intake of milk five times per week acts as a moderator of the connection between sleep quality and depression; **(E)** intake of soybean five times per week acts as a moderator of the connection between sleep quality and anxiety; **(F)** intake of soybean five times per week acts as a moderator of the connection between sleep quality and depression; **(G)** intake of fish/meat/eggs five times per week acts as a moderator of the connection between sleep quality and anxiety; **(H)** intake of fish/meat/eggs five times per week acts as a moderator of the connection between sleep quality and depression; **(I)** whether doing outdoor exercise for at least 30 min daily acts as a moderator of the connection between sleep quality and anxiety; **(J)** whether doing outdoor exercise for at least 30 min daily acts as a moderator of the connection between sleep quality and depression. Low sleep quality means one standard deviation below the mean of sleep quality; high sleep quality means one standard deviation above the mean of sleep quality. *p < 0.05; **p < 0.01; ***p < 0.001.

As depicted in Figure 6, simple slope analysis showed that: for older adults with bodily pains over the past years, sleep quality had a strong positive effect on psychological states (*p* < 0.001) (Figure 6A), whereas this effect was non-significant for those with no bodily pains over the past years(*p* > 0.05) (Figure 6B); when older adults could not or occasionally could not intake milk (*p* < 0.001) (Figure 6C), soybean (*p* < 0.01) (Figure 6E), or fish/poultry/eggs (*p* < 0.001) (Figure 6G) to reach five times per week, or when older adults could not do outdoor exercise for at least 30 min daily (*p* < 0.001) (Figure 6I), sleep quality had a significant positive predictive effect on psychological states; when older adults could intake milk (*p* < 0.05) (Figure 6D), soybean (*p* < 0.001) (Figure 6F), or fish/poultry/eggs (*p* < 0.01) (Figure 6H) to reach five times a week, or do outdoor exercise for at least 30 min daily (*p* < 0.05) (Figure 6J), the positive predictive effect of sleep quality on psychological states was diminished. Supplementary Figure S2 shows simple slope plots for the remaining factors.

Furthermore, analyses of conditional indirect effects showed (Table 3) that the mediating effects of anxiety on the association between sleep quality and frailty was positively moderated by whether there have been bodily pains over the past years (*B* = 0.031, Boot 95%CI [0.005, 0.069]), and negatively moderated by the intake of milk (*B* = −0.035, Boot 95%CI [−0.0855, −0.003]), soybean, and fish/meat/eggs five times per week (*B* = −0.031, Boot 95%CI [−0.075, −0.001]), and whether doing outdoor exercise for at least 30 min daily (*B* = −0.050, Boot 95%CI [−0.122, −0.005]). Specifically, when there has been pain in any part of the body over the past years, the conditional indirect effect of anxiety (*B* = 0.039, Boot 95%CI [0.006, 0.083]) was statistically significant. When older adults could not or occasionally could not intake milk (*B* = 0.054, Boot 95%CI [0.008, 0.116]), soybean (*B* = 0.052, Boot 95%CI [0.007, 0.110]), or fish/poultry/eggs (*B* = 0.072, Boot 95%CI [0.011, 0.158]) to reach five times per week, or when older adults could not do outdoor exercise for at least 30 min daily (*B* = 0.051, Boot 95%CI [0.0089, 0.120]), the conditional indirect effect of anxiety was statistically significant. When older adults intake milk(*B* = 0.019, Boot 95%CI [0.002, 0.041]), soybean (*B* = 0.021, Boot 95%CI [0.003, 0.047]), or fish/poultry/eggs (*B* = 0.022, Boot95% CI [0.003, 0.049]) to reach five times per week, or did outdoor exercise for at least 30 min daily (*B* = 0.020, Boot 95%CI = [0.004, 0.041]), the conditional indirect effect of anxiety were still significant but diminished. However, these same moderators failed to demonstrate significant moderating effects on the mediating effect of depression. Furthermore, no significant moderated mediating effects were observed for BMI, fall history, recent weight change, daily meal frequency, whether drinking water reached 1,200 mL daily, smoking history, and drinking history (Supplementary Table S2).

**Table 3:** The conditional indirect effects of sleep quality on frailty under different conditions of pain, milk, soybean, fish/meat/eggs, and exercise.

Moderator	Effect	Boot SE	Boot 95%CI
Pain (sleep quality → anxiety → frailty)			
No	0.008	0.008	−0.002, 0.027
Yes	0.039	0.020	0.006, 0.083
Moderated mediation index	0.031	0.017	0.005, 0.069
Pain (sleep quality → depression → frailty)			
No	−0.003	0.006	−0.016, 0.008
Yes	0.029	0.017	−0.001, 0.064
Moderated mediation index	0.032	0.019	−0.001, 0.072
Milk (sleep quality → anxiety → frailty)			
Sometimes or not	0.054	0.027	0.008, 0.116
Always	0.019	0.010	0.002, 0.041
Moderated mediation index	−0.035	0.0208	−0.0855, −0.003
Milk (sleep quality → depression → frailty)			
Sometimes or not	0.042	0.024	−0.0002, 0.095
Always	0.009	0.007	−0.002, 0.025
Moderated mediation index	−0.033	0.020	−0.079, 0.0002
Soybean (sleep quality → anxiety → frailty)			
Sometimes or not	0.052	0.026	0.007, 0.110
Always	0.021	0.011	0.003, 0.047
Moderated mediation index	−0.031	0.019	−0.075, −0.001
Soybean (sleep quality → depression → frailty)			
Sometimes or not	0.038	0.021	0.0003, 0.084
Always	0.011	0.008	−0.0004, 0.028
Moderated mediation index	−0.026	0.017	−0.065, 0.001
Fish/meat/eggs (sleep quality → anxiety → frailty)			
Sometimes or not	0.072	0.037	0.011, 0.158
Always	0.022	0.012	0.003, 0.049
Moderated mediation index	−0.050	0.030	−0.122, −0.005
Fish/meat/eggs (sleep quality → depression → frailty)			
Sometimes or not	0.063	0.036	−0.001, 0.139
Always	0.012	0.008	−0.0003, 0.029
Moderated mediation index	−0.051	0.031	−0.119, 0.001
Exercise (sleep quality → anxiety → frailty)			
Sometimes or not	0.051	0.029	0.0089, 0.120
Always	0.020	0.010	0.004, 0.041
Moderated mediation index	−0.032	0.022	−0.089, −0.002
Exercise (sleep quality → depression → frailty)			
Sometimes or not	0.042	0.024	−0.001, 0.092
Always	0.009	0.006	−0.001, 0.024
Moderated mediation index	−0.033	0.020	−0.079, 0.001

Pain: bodily pains; milk, soybean, and fish/meat/eggs: intake of milk, soybean, and fish/meat/eggs five times per week; exercise: whether doing outdoor exercise for at least 30 min daily.

## 4 Discussion

To our knowledge, this is the first study to simultaneously examine the parallel mediating effects of psychological states (anxiety and depression) in the relationship between sleep quality and frailty in older adults, and further reveals the moderating effects of bodily pain, dietary habits (such as the consumption of milk, soybeans, fish, poultry, and eggs), and outdoor physical activity on the “sleep quality → anxiety” pathway.

Our findings align with international research on partial mediation and moderation effects (Zhang et al., 2024; Duncan et al., 2021; Sheffler et al., 2023; Zhang et al., 2022). Previous studies have established depression as a mediator in the sleep-frailty relationship (Zhang et al., 2024). Others have demonstrated physical activity’s moderating effect on the association between sleep quality and psychological distress (Duncan et al., 2021; Sheffler et al., 2023; Zhang et al., 2022). However, our study is the first to incorporate others modifiable factors like bodily pain and dietary habits into a unified theoretical framework. The findings not only represent a significant advancement in the integration of these mechanisms, but also provide new theoretical perspectives for this area of research.

The biological pathways underpinning these connections are multifaceted. Sleep, as an important physiological process in the human body, is critical for the maintenance of hormonal homeostasis, energy metabolism, and cardiovascular function. However, factors such as decreased melatonin secretion associated with aging significantly increase the susceptibility to sleep disorders in the elderly population (Pappas and Miner, 2022). Aberrant sleep patterns, including excessively long or short sleep duration, daytime sleepiness, sleep apnea, and prolonged sleep latency, can disrupt the balance of inflammatory cytokines, adipokines, and hormones, thereby elevating frailty risk (Pourmotabbed et al., 2020). At the same time, impaired sleep quality also constitutes the pathophysiological basis of frailty by inhibiting the protein synthesis pathway and the catabolic processes in skeletal muscle, impairing muscle mass and function (Yuan and Larsson, 2023; Nascimento et al., 2019). Furthermore, chronically poor sleep quality adversely impacts emotional well-being in older adults, heightening anxiety about fatigue and fostering negative affective states like depression. The state of anxiety itself may lead to deterioration of health and become a frailty influence, while depression can lead to withdrawal from daily activities due to low mood and psychomotor retardation, thereby increasing frailty vulnerability.

This study also provides the first evidence that bodily pain, specific dietary habits (intake frequency ≥5 times/week for milk, soybean, and fish/meat/eggs), and daily outdoor exercise duration (≥30 min) significantly moderated the strength of the association of the mediating pathway “sleep quality → anxiety,” consequently influencing its indirect effect on frailty. Firstly, the presence of bodily pain significantly amplified the association between poor sleep quality and anxiety symptoms. This finding is consistent with the widespread evidence that pain is often accompanied by anxiety and exacerbates adverse health outcomes (Khan et al., 2020; Bair et al., 2003; Gorczyca et al., 2013; Lee et al., 2020; Xu et al., 2022; Irwin et al., 2012; Amtmann et al., 2015). The underlying mechanism may involve a vicious cycle of pain interfering with sleep maintenance and sleep deprivation exacerbating pain perception, which together lead to patients’ reduced energy, diminished physical activity, and increased anxiety about poor health prognosis. Secondly, higher intake frequencies of soybean and fish/meat/egg (≥5 times/week) may mitigate the negative effects of poor sleep quality on anxiety symptoms. The rich proteins and vitamins in soybeans, as well as the high-quality protein, long-chain omega-3 polyunsaturated fatty acids, and vitamin D provided by fish/meat/eggs (Shahidi and Ambigaipalan, 2018; Benedik, 2022), may attenuate the transition from sleep problems to anxiety by participating in sleep regulation (Chen et al., 2022; Abboud, 2022), maintaining neurological function, and alleviating anxiety (Patrick and Ames, 2015; Kołodziej et al., 2023). Milk intake, on the other hand, presents complex effects. Although its richness in nutrients such as vitamin D, calcium, and B vitamins has been suggested to potentially improve sleep (Komada et al., 2020; Xu et al., 2023), and its non-consumption may be associated with poor mental state (Tseng et al., 2019). However, the UK Biobank study reported an association of higher milk intake with poorer sleep quality and more psychiatric symptoms, suggesting that the direction of its modulation of the sleep-psychiatric association needs to be further clarified (Hepsomali and Groeger, 2021). In addition, ≥30 min of daily outdoor exercise demonstrated a significant attenuating effect on the exacerbating influence of poor sleep quality on anxiety symptoms. This observation is substantiated by extensive empirical evidence (Vanderlinden et al., 2020; Chen et al., 2009; Saeed et al., 2019; de Oliveira et al., 2019). A randomized controlled trial in Australia further confirmed that exercise intervention improved psychological status in people with poor sleep quality (Duncan et al., 2021).

In summary, this study provides novel mechanistic insights and actionable intervention targets for understanding and mitigating the progression from sleep problems to frailty through multidimensional and modifiable approaches. Previous studies have shown that identifying anxiety symptoms in older adults with sleep disturbances may aid in preventing frailty progression, particularly during the pre-frailty stage (Borges et al., 2022; Tan et al., 2023). Meanwhile, alleviating depressive symptoms in older chronic kidney disease patients with comorbid sleep disorders could help delay frailty onset (Wang et al., 2024). Notably, anxiety and depression often coexist and psychological interventions to improve anxiety states (e.g., cognitive behavioral therapy) may indirectly delay frailty by optimizing sleep quality and reducing depressive symptoms (Wu et al., 2024). Therefore, community-based screening for anxiety in older adults with sleep disorders could represent a cost-effective strategy for frailty prevention. Simultaneously, relevant policies should be formulated to help older adults maintain healthy lifestyles, such as engaging in regular physical activity, adhering to appropriate dietary habits, and actively managing chronic diseases. Furthermore, the accessibility and sustainability of interventions should be considered to ensure their broad promotion and application at the community level, thereby bringing tangible health benefits to more older adults with sleep disturbances. Consequently, future research are needed to implement personalized comprehensive intervention strategies.

This study still had some limitations. Firstly, the cross-sectional nature of our design limits the ability to infer causal connections among the variables. Future longitudinal cohort studies are needed to better establish temporal sequences and causal pathways. Second, the use of retrospective self-report instrumental measures may introduce recall bias. Incorporating objective assessment methods such as actigraphy or biomarker-based evaluations in future research would help mitigate this limitation. Thirdly, the participants were obtained from a single region, which may affect the generalizability of the findings. Replication in more diverse populations, including rural/urban settings and different ethnic groups, is warranted. Additionally, despite adjusting for several covariates, residual confounding from unmeasured variables (e.g., comorbidities, medication use, or social support) cannot be ruled out. Finally, some baseline characteristics were unevenly distributed across groups, a limitation exacerbated by the modest sample size. Future studies with larger, multi-center samples and prospective designs are necessary to validate and extend our findings.

## 5 Conclusion

In conclusion, our findings highlight that sleep quality is not only a direct determinant of frailty but also exerts its impact indirectly through anxiety and depression. The extent of this mediation varies among older adults, influenced by differences in physiological conditions, dietary habits, and lifestyles, addressing these psychological factors may represent a pivotal intervention point in preventing or delaying frailty, particularly when enhancements in sleep quality are unattainable. Thus, future interventions should include routinely assessing sleep quality, anxiety and depression in geriatrics and primary care to identify at-risk individuals earlier. Additionally, integrated strategies addressing chronic pain, nutrition, and physical activity are essential. Further clinical trials evaluating combined interventions targeting sleep, psychological states, chronic pain, nutritional habits, and physical activity are warranted to develop effective frailty prevention programs.

## Data Availability

The original contributions presented in the study are included in the article/Supplementary material, further inquiries can be directed to the corresponding authors.
