# Supplementary material for: Psychological states mediate the relationship between sleep quality and frailty among older adults
Source: Front Psychol. 2025 Nov 12;16:1691997. doi: 10.3389/fpsyg.2025.1691997 (PMC12646993; doi:10.3389/fpsyg.2025.1691997)
Supplement: Supplementary file 1 [file Supplementary_file_1.docx]

**Supplementary materials**

**Figure Legends:**

**Figure S1. The moderated mediation path diagram.** Note: A. The moderating effect of BMI on the mediation model; B. The moderating effect of falls history on the mediation model; C. The moderating effect of recent weight change on the mediation model; D. The moderating effect of daily meal frequency on the mediation model; E. The moderating effect of whether drinking water reaching 1200ml daily on the mediation model; F. The moderating effect of smoking history on the mediation model; G. The moderating effect of drinking history on the mediation model. c'= direct effects .*：p<0.05; **：p<0.01; ***p<0.001. ⊗: The conditional indirect effects are not valid.

**Figure S2. The simple slope plots with the multidimensional factors as moderators.** Note: A. BMI acts as a moderator of the connection between sleep quality and anxiety; B. BMI acts as a moderator of the connection between sleep quality and depression; C: Falls history acts as a moderator of the connection between sleep quality and anxiety; D. Falls history acts as a moderator of the connection between sleep quality and depression; E. Recent weight change acts as a moderator of the connection between sleep quality and anxiety; F. Recent weight change acts as a moderator of the connection between sleep quality and depression; G. Daily meal frequency acts as a moderator of the connection between sleep quality and anxiety; H. Daily meal frequency acts as a moderator of the connection between sleep quality and depression; I. Whether drinking water reaching 1200ml daily acts as a moderator of the connection between sleep quality and anxiety; J. Whether drinking water reaching 1200ml daily acts as a moderator of the connection between sleep quality and depression. K. Smoking history acts as a moderator of the connection between sleep quality and anxiety. L. Smoking history acts as a moderator of the connection between sleep quality and depression. M. Drinking history acts as a moderator of the connection between sleep quality and anxiety. N. Drinking history acts as a moderator of the connection between sleep quality and depression. Low sleep quality means one standard deviation below the mean of sleep quality; high sleep quality means one standard deviation above the mean of sleep quality. *: p<0.05; **: p<0.01; ***p<0.001.

**Tables**

**Table S1. Collinearity testing.**

**Table S2. The conditional indirect effects of sleep quality on frailty under different conditions of BMI, fall, weight, meal frequency, water, smoking, and drinking.**

**Table S1. Collinearity testing.**

| **Variable** | **VIF** | **1/VIF** |
| --- | --- | --- |
| age | 1.143 | 0.875 |
| gender | 1.423 | 0.703 |
| marriage | 1.225 | 0.816 |
| BMI | 1.02 | 0.980 |
| pain | 1.144 | 0.874 |
| fall | 1.12 | 0.893 |
| weight | 1.355 | 0.738 |
| eat | 1.411 | 0.709 |
| milk | 1.871 | 0.534 |
| soybean | 1.792 | 0.558 |
| fish | 1.479 | 0.676 |
| water | 1.544 | 0.647 |
| exercise | 1.169 | 0.855 |
| smoke | 1.449 | 0.690 |
| drink | 1.424 | 0.702 |
| PSQI | 1.171 | 0.854 |
| anxiety | 2.012 | 0.497 |
| depression | 2.044 | 0.489 |
| Mean VIF | 1.415 | |

*Note:* pain: bodily pains; fall: falls history; weihgt: recent weight change; meal frequency: daily meal frequency; milk, soybean, and fish/meat/eggs: condition of intake of milk, soybean, and fish/meat/eggs 5 times per week; water: whether drinking water reaching 1200ml daily; exercise: whether doing outdoor exercise for at least 30 minutes daily; smoking: smoking history; drinking: drinking history; .PSQI: sleep quality; HADS-A: anxiety; HADS-D: depression; FRAIL: frailty.

**Table S2. The conditional indirect effects of sleep quality on frailty under different conditions of BMI, fall, weight, meal frequency, water, smoking, and drinking.**

| **Moderator** | **B** | **BootSE** | **Boot95%Cl** |
| --- | --- | --- | --- |
| BMI(sleep quality→anxiety →frailty) |  |  |  |
| BMI-1SD | 0.021 | 0.012 | 0.002,0.049 |
| BMI | 0.029 | 0.014 | 0.005,0.059 |
| BMI+1SD | 0.036 | 0.019 | 0.006,0.079 |
| moderated mediation index | 0.008 | 0.007 | -0.003,0.024 |
| BMI(sleep quality→depression →frailty) |  |  |  |
| BMI-1SD | 0.012 | 0.008 | -0.001,0.031 |
| BMI | 0.018 | 0.010 | -0.0002,0.041 |
| BMI+1SD | 0.024 | 0.015 | -0.0002,0.059 |
| moderated mediation index | 0.006 | 0.006 | -0.002,0.022 |
| fall(sleep quality→anxiety →frailty) |  |  |  |
| sometimes or not | 0.023 | 0.013 | 0.002,0.051 |
| always | 0.049 | 0.025 | 0.006,0.104 |
| moderated mediation index | 0.026 | 0.017 | -0.002,0.065 |
| fall(sleep quality→depression →frailty) |  |  |  |
| sometimes or not | 0.013 | 0.008 | 0.0002,0.033 |
| always | 0.033 | 0.020 | 0.0003,0.079 |
| moderated mediation index | 0.020 | 0.016 | -0.006,0.057 |
| weight(sleep quality→anxiety →frailty) |  |  |  |
| no | 0.035 | 0.017 | 0.005,0.074 |
| yes | 0.019 | 0.012 | 0.001,0.048 |
| moderated mediation index | -0.016 | 0.013 | -0.048,0.004 |
| weight(sleep quality→depression →frailty) |  |  |  |
| no | 0.027 | 0.015 | 0.001,0.061 |
| yes | 0.011 | 0.009 | -0.002,0.034 |
| moderated mediation index | -0.016 | 0.012 | -0.045,0.002 |
| meal frequency(sleep quality→anxiety →frailty) |  |  |  |
| sometimes or not | 0.025 | 0.014 | 0.003,0.057 |
| always | 0.032 | 0.016 | 0.005,0.069 |
| moderated mediation index | 0.008 | 0.011 | -0.014,0.031 |
| meal frequency(sleep quality→anxiety →frailty) |  |  |  |
| sometimes or not | 0.016 | 0.011 | -0.0001,0.041 |
| always | 0.019 | 0.012 | 0.0002,0.047 |
| moderated mediation index | 0.003 | 0.009 | -0.014,0.024 |
| water(sleep quality→anxiety →frailty) |  |  |  |
| sometimes or not | 0.041 | 0.020 | 0.007,0.086 |
| always | 0.012 | 0.009 | -0.001,0.034 |
| moderated mediation index | -0.029 | 0.017 | -0.067,-0.003 |
| water(sleep quality→depression →frailty) |  |  |  |
| sometimes or not | 0.026 | 0.015 | -0.0003,0.059 |
| always | 0.008 | 0.007 | -0.002,0.026 |
| moderated mediation index | -0.018 | 0.013 | -0.048,0.001 |
| smoking(sleep quality→anxiety →frailty) |  |  |  |
| no | 0.029 | 0.014 | 0.004,0.060 |
| yes | 0.025 | 0.021 | -0.003,0.076 |
| moderated mediation index | -0.004 | 0.017 | -0.04,0.033 |
| smoking(sleep quality→depression →frailty) |  |  |  |
| no | 0.018 | 0.011 | 0.0001,0.042 |
| yes | 0.018 | 0.017 | -0.007,0.059 |
| moderated mediation index | -0.001 | 0.016 | -0.033,0.033 |
| drinking(sleep quality→anxiety →frailty) |  |  |  |
| no | 0.029 | 0.014 | 0.006,0.060 |
| yes | 0.025 | 0.021 | -0.004,0.078 |
| moderated mediation index | -0.004 | 0.018 | -0.041,0.036 |
| drinking(sleep quality→depression →frailty) |  |  |  |
| no | 0.017 | 0.010 | 0.0005,0.041 |
| yes | 0.020 | 0.018 | -0.006,0.063 |
| moderated mediation index | 0.002 | 0.015 | 0.027,0.037 |

*Note:* fall: falls history; weight: recent weight change; meal frequency: daily meal frequency; water: whether drinking water reaching 1200ml daily; smoking: smoking history; drinking: drinking history.
