# Supplementary figures and images for: Psychological states mediate the relationship between sleep quality and frailty among older adults
Source: Front Psychol. 2025 Nov 12;16:1691997. doi: 10.3389/fpsyg.2025.1691997 (PMC12646993; doi:10.3389/fpsyg.2025.1691997)

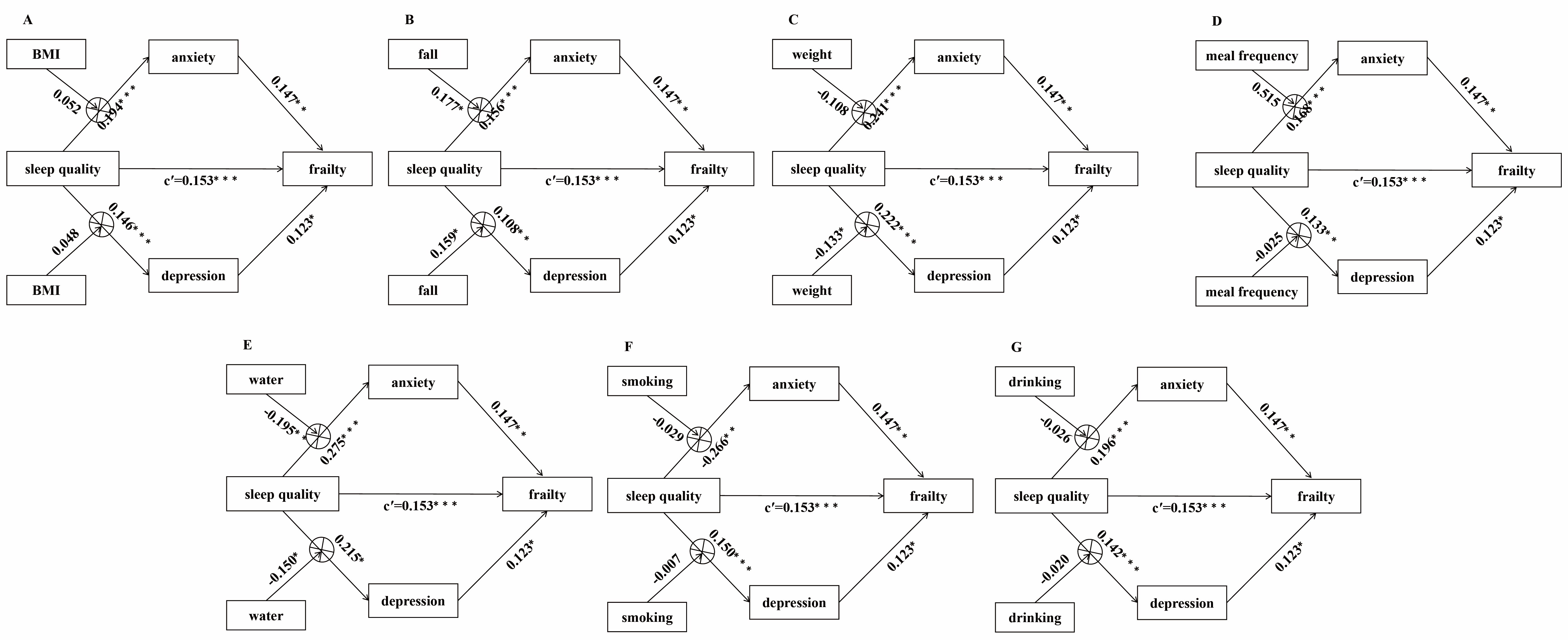

Supplement: Supplementary file 2 [file Image_1.jpeg]

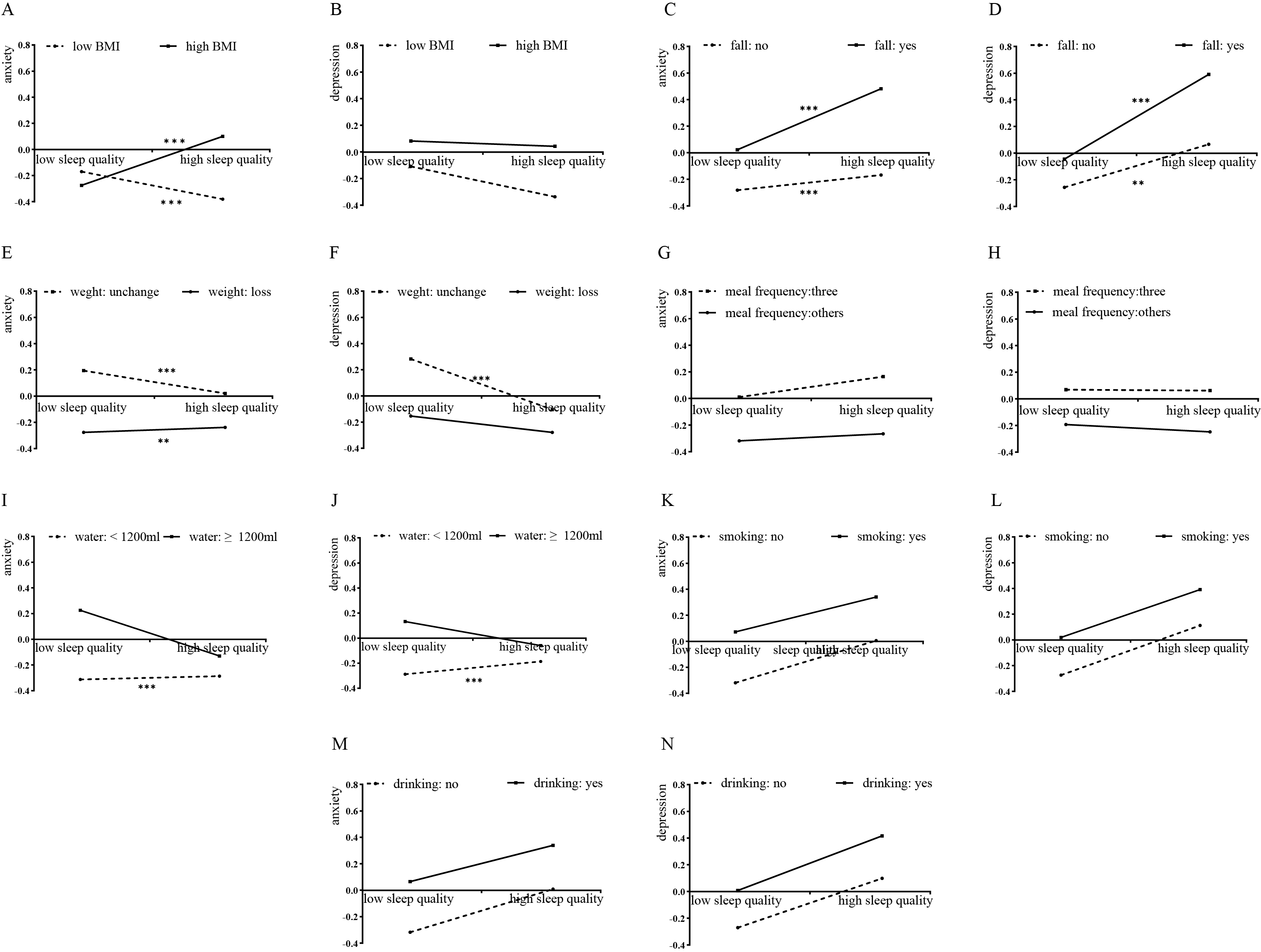

Supplement: Supplementary file 3 [file Image_2.tif]
